# 560. Superficial Burn Injuries Associated with Higher Rates of Non-melanoma Skin Cancers: A Population-based Retrospective Study

**DOI:** 10.1093/jbcr/irag033.300

**Published:** 2026-04-06

**Authors:** Vincent Yang, Lynchi Nguyen, Juquan Song, Steven E Wolf, Amina El Ayadi

**Affiliations:** Shriners Burn Center - Galveston, Galveston, TX; University of Texas Medical Branch John Sealy School of Medicine, Galveston, TX; University of Texas Medical Branch, Galveston, TX; Division of Burn and Trauma Surgery, Department of Surgery, University of Texas Medical Branch, Galveston, Galveston, TX; Division of Burn and Trauma Surgery, Department of Surgery, University of Texas Medical Branch, Galveston, Galveston, TX

## Abstract

**Introduction:**

Burns are classified by dermal involvement (partial- vs full-thickness). Evidence on long-term skin cancer risk in burn survivors is mixed: some cohort studies show no increase, while case reports and mechanistic data suggest elevated basal cell carcinoma (BCC) and squamous cell carcinoma (SCC). We aimed to assess how risk for non-melanoma skin cancer (NMSC), including carcinoma in situ (CIS), BCC, and SCC, varies by burn depth (1st, 2nd, or 3rd degree) and inform clinical counseling.

**Methods:**

A retrospective cohort study using TriNetX (102 healthcare organizations) identified over 131 000 patients with 1st, 2nd, or 3rd degree burns diagnosed ≥10 years ago and assigned them to depth subcohorts. Propensity score matching balanced demographics and comorbidities. Outcomes ≥1 year post-burn diagnosis were assessed. Risk ratios (RR) and 95% confidence intervals (CI) were calculated.

**Results:**

NMSC risk was higher in patients with 1st (RR = 2.171, CI: 1.457–3.237) and 2nd degree (2.525, 1.753–3.637) vs. 3rd degree burns. Upon stratification by cancer subtype: CIS risk was increased in 1st degree vs. 2nd degree (2.142, 1.136–4.038) and 3rd degree burns (2.099, 0.989–4.455); BCC risk was significantly higher in 1st degree (2.428, 1.462–4.032) and 2nd degree (1.800, 1.105–2.932) vs. 3rd degree; SCC risk was significantly increased only in 1st degree vs. 3rd degree (2.154, 1.117–4.155).

**Conclusions:**

Superficial burns carry higher long-term NMSC risk than full-thickness injuries, with elevated risk particularly for BCC and CIS. This pattern fits known biology because superficial injuries preserve keratinocytes and adnexal structures, whereas third-degree burns leave residual disturbed dermal cells. Burn depth should inform survivorship counseling and guide studies that include burn size, etiology, and anatomical location.

**Applicability of Research to Practice:**

Although risk counseling exists for burn survivors, our data clarifies depth-related differences and supports standardized counseling and routine surveillance across all severities. Survivors of superficial burns should receive focused education on BCC and CIS risk, self-exams of scars and nearby skin, UV protection, and clear thresholds for dermatology visits. Adding burn depth to survivorship care plans and referral pathways can mitigate the false narrative that more superficial burns carry less risk than deeper burns, leading to earlier detection and better long-term outcomes.

**Funding for the study:**

N/A.

